# Electroacupuncture Upregulates HIF-1*α* and SOX9 Expression in Knee Osteoarthritis

**DOI:** 10.1155/2021/2047097

**Published:** 2021-11-01

**Authors:** Jie Tong, Chengyuan Deng, Guanghua Sun, Jun Zhou, Peirui Zhong, Tiantian Wang, Ye Zeng, Qi Wu, Yuan Liao, Fu Luo, Ting Peng, Ying Liao

**Affiliations:** ^1^Department of Rehabilitation, The First Affiliated Hospital of University of South China, Hengyang 421001, Hunan, China; ^2^Rehabilitation Laboratory, The First Affiliated Hospital of University of South China, Hengyang 421001, Hunan, China; ^3^Rehabilitation Medicine Center, The First Affiliated Hospital of University of South China, Hengyang 421001, Hunan, China; ^4^Department of Anatomy, Hunan Traditional Chinese Medical College, Zhuzhou 412000, Hunan, China

## Abstract

Electroacupuncture (EA) has been clinically used in knee osteoarthritis broadly and proved to be effective than other therapies with fewer side effects; however, the mechanism of electroacupuncture to work on cartilage remains unclear. In this study, we aimed to evaluate the effect of EA treatment on cartilage and the relationship between EA and proteins such as HIF-a and SOX9. EA (dilatational wave, 3–15 HZ, 1 mA) has been applied to bilateral Zusanli (ST36), Xuehai (SP10), Taixi (KI3), and Yanglingquan (GB34) of rats. Results showed that the cartilage of the knee osteoarthritis group had obvious damage and fissure formation while the EA group showed that the cartilage destruction was generally milder. In addition, the protein expression levels of HIF-1*α*, and chondrogenic markers such as Sox9, and ACAN in the electroacupuncture group were higher than those in the ACLT group. Also, the extracellular matrix protein expression levels of MMP13 and ADAMTS5 were decreased in the EA group. These findings indicate that EA could alleviate the severity of knee osteoarthritis, and HIF-a and SOX9 may closely attribute to the treatment.

## 1. Introduction

Osteoarthritis (OA) is the most common degenerative joint disease and the leading cause of physical disability [1]. Knee osteoarthritis affects approximately 265 million people worldwide and was estimated in 2017 to account for 8.3 million years lived with disability [2]. However, despite its prevalence and severity, few safe and effective treatments are available for patients with osteoarthritis so far, due to poor understanding of disease mechanisms [3]. OA is characterized by cartilage degeneration, bone remodeling [4], osteophytes, synovitis, and sclerosis [5]. Damaged cartilage has limited capacity of regeneration and repairment for its avascular and nerveless structure, resulting in progressive total joint destruction [6]. Adult articular cartilage consists of chondrocytes and extracellular matrix (ECM), such as collagens and proteoglycans including aggrecan (ACAN) [7]. In healthy joints, balance between chondrocyte synthesis of anabolic factors such as type II collagen and ACAN and catabolic factors such as matrix metalloprotease 13 (MMP13) and a disintegrin and metalloproteinase with thrombospondin motifs (ADAMTS) maintains the homeostasis of articular cartilage [8]. Respectively, ACAN can be cleaved by ADAMTS, which is a family of aggrecanases [8]. In addition, collagen could be degraded by MMPs, especially MMP13 [9]. So, reduction of catabolic enzymes may be sufficient to inhibit OA disease progression, particularly at early stages of disease. Additionally, Sry-related HMG box-9 (SOX9), a member of the chondrogenesis family, can suppress excessive *β*-catenin activity, which indirectly inhibits MMP13 expression and slows down the progression of OA [10]. SOX9 binds to genes encoding major cartilaginous matrix proteins such as ACAN and type II collagen-*α*1 (Col2a1) and regulates their expression [11]. Hypoxia‐inducible factor (HIF) is the most direct or unique regulatory factor that has been found to acclimatize tissues or cells to hypoxia [12] and comprises a tightly regulated HIF-*α* subunit (HIF-1*α* and HIF-2*α*) [13]. HIF-1a regulates mitochondrial dysfunctions via mitophagy in the damaged chondrocytes and then inhibits the degeneration of cartilage [14].

Electroacupuncture (EA) refers to the application of a pulsating electrical current to acupuncture needles for acupuncture point stimulation [15]. As a traditional Chinese medicine, EA effectively alleviates cartilage degeneration [16], thus remarkably improving the life quality of OA patients [17], and has fewer side effects compared to pharmacotherapy [18]. Studies have shown that the progression of ACLT-induced posttraumatic OA has been delayed by electroacupuncture via reducing the expression of IL-1*β* and MMP3, while the mechanism has been unambiguous [19].

To date, the mechanism that EA improves KOA has not been fully illustrated. Research, recently, reported that EA may have an anti-inflammatory effect on OA [20–22]. However, the role of EA in alleviating cartilage degradation and subchondral bone remolding in OA remains ambiguous. Thus, in this study, we established OA rats by anterior cruciate ligament transection (ACLT) to mimic instability of joint, which leads to the initiation and development of posttraumatic OA [23]. We aimed to determine the effectiveness of EA in terms of cartilage protection and the changes in the subchondral bone using safranin-O staining and micro-CT scanning to see if EA could act as a stimulating factor for cartilage regeneration and subchondral bone preservation. Furthermore, we investigated the potential functional role of HIF-1a SOX9 in OA pathology and the underlying molecular mechanisms of EA in alleviating the development of OA.

## 2. Materials and Methods

### 2.1. Ethical Statement

Three-month-old male Sprague Dawley rats were purchased from Hunan SJA Laboratory Animal Co., LTD., and were housed in a 12/12-hour light/dark cycle in a cage with a relative humidity of 55 ± 5% at 24 ± 2°C RT. The supplies of water and food were not restricted. All surgical and treatment procedures have been approved by the ethics committee of the First Affiliated Hospital of University of South China (Reference number 20160220) and implemented in accordance with the ethical standards contained in the committee's charter.

### 2.2. Grouping

The 24 rats were randomly divided into three groups (8 rats/group): a control group (sham-operation group); ACLT group; and EA group (ACLT group receiving EA treatment).

### 2.3. ACLT-Induced Osteoarthritis Rats

Specific procedures of ACLT surgery were as follows [24]: (1) the rats were anesthetized with pentobarbital sodium intraperitoneally (40 mg/kg) and fixed on a dissection platform, and their bilateral knees were shaved and disinfected using 75% alcohol; (2) under general anesthesia, incisions were made at the medial side of knee joints to expose the cavity, and the anterior cruciate ligament was cut. In this process, care was taken to avoid cartilage damage; and (3) a drawer test is performed to confirm whether the modeling is successfully established. Only positive results can achieve hemostasis and suture. In addition, the control group exposed the cavity, but the anterior cruciate ligament was not cut.

### 2.4. EA Treatment

12 weeks following the ACLT surgery, the EA group were fixed on the board in the supine position for treatment. A disposable sterile acupuncture needle (length:25 mm, diameter: 0.25 mm; Huatuo, Suzhou Medical Equipment Factory, China) was inserted into rats in the EA group at a depth of 3 mm at bilateral Zusanli (ST36, located at the anterior tibia muscle, about 3 mm below the knee joint), Xuehai (SP10, located at the bulge of the vastus medialis muscle, about 2 B-cun superior to the medial end of the base of the patella), Taixi (KI3, located between the medial malleolus and the calcaneus.), and Yanglingquan (GB34, located at the side of the crus, at the bottom and under the head of fibula in rats). The mode of the electroacupuncture apparatus (Suzhou Medical Supplies Factory) is set to sparse-dense wave, delivering frequency is at 3/15 pulse, and intensity is at 1 mA. The apparatus runs once a day, 30 minutes each time, 5 days a week for 12 weeks. Alcohol (75%) will be used to routinely disinfect the local skin at the acupuncture points.

### 2.5. Histopathology Analysis

After intervention, the left tibial plateaus were harvested under general euthanasia, and all tissues were washed 3 times with phosphate-buffered saline, decalcified with 4% paraformaldehyde for 48 h, dehydrated by ethanol, placed in an incubator, and then, embedded in paraffin. The left tibial plateaus were serially sectioned into 5 *μ*m slices and stained with HE and safranin-O. The pathological sections were evaluated by two independent, blinded observers according to the Mankin scoring criteria. The evaluation was based on the modified Mankin scoring system, which includes (1) structural integrity (6 points scale where 0 = normal and 6 = completely disorganized); (2) cell density (3 points scale where 0 = normal and 3 = hypo cellular); (3) safranin-O staining (4 points scale where 0 = normal and 4 = no staining); and (4) tidemark integrity (binary scale where 0 = normal and 1 = disrupted).

### 2.6. Micro-CT and 3D Imaging Analysis

The right tibia platform was scanned via the Shanghai synchrotron radiation source BL13W1 X-ray station and imported into the VG Studio MAX2.3 system for 3D imaging. We observe and use the software to the cartilage, and subchondral bone structure changes were observed and calculated for the following values: ratio of subchondral bone volume/total volume ratio (BS/TV), ratio of bone surface area/bone volume ratio (BS/BV), trabecular bone (column structure), average thickness (TbTh), average number of trabeculae per unit length (TbN), average distance between trabeculae (column structure) (TbSp), and other parameters. The instrument parameters were set as follows: 25 kev energy, 0.75 m distance, 8 ms exposure time, and 9 *μ*m spatial resolution.

### 2.7. Western Blot Analysis

Knee articular cartilage tissue from the femoral condyle was homogenized in a lysate containing protease inhibitors, and proteins extracted were analyzed by SDS-PAGE (10% separation gel and 4.8% concentrated gel) electrophoresis. After blocking the 5% delipidated protein, 0.05 g sample was taken and ground in liquid nitrogen. Primary antibodies HIF-1*α* (ab1, 5 *μ*g/ml, Abcam); SOX9 (67439-1-Ig, 1 : 1000, protein tech); MMP13 (ab39012, 1 : 1000, Abcam); ADAMTS5 (ab41037, 1 : 500, Abcam), ACAN (ab3778, 1 : 1000, Abcam); and actin (60008-1-Ig, 1 : 5000, protein tech) were added. After washing, secondary antibody HRP goat anti-mouse IgG (SA00001-1, 1 : 5000, protein tech) and HRP goat anti-rabbit IgG (SA00001-2, 1 : 6000, American protein tech) were added. Immunocomplexes were visualized with chemiluminescence using an ECL kit. Data given above were analyzed by quantity one, a professional gray-level analysis software.

### 2.8. Statistical Analysis

All data are presented as mean ± standard deviations (SDs). Comparisons between groups were determined by one-way analysis for variance (ANOVA) followed by Tukey's post hoc test in SPSS 24.0 (IBM SPSS statistic, USA), and *P* < 0.05 was considered as statistically significant.

## 3. Result

### 3.1. Effect of EA on the Morphology and Structure of Articular Cartilage in OA Rats

**Figure 1:**
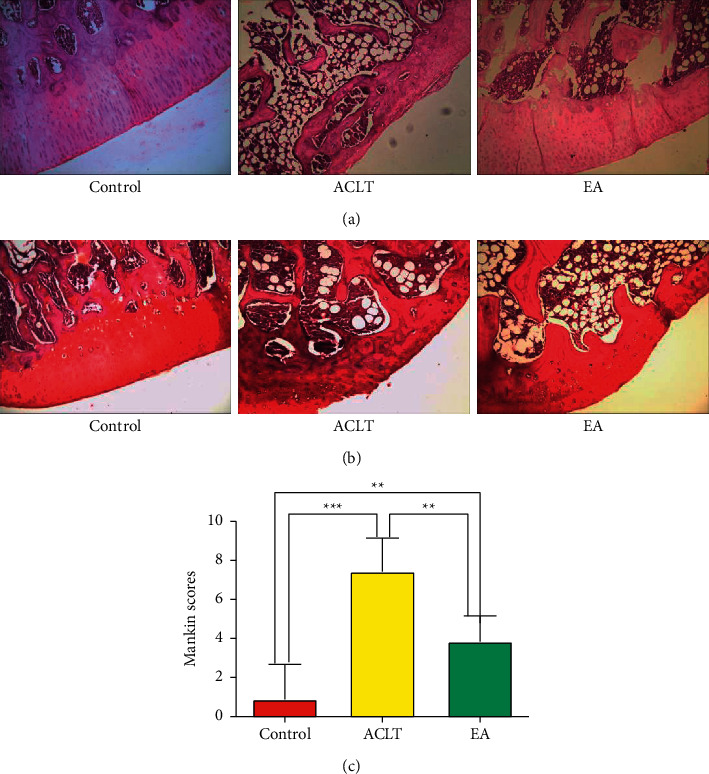
(a), (b) Representative images (∗ 100 magnification). Control, ACLT, and EA, respectively, represent the control group, ACLT group, and EA group. (c) Modified Mankin scores (maximum = 14). ^*∗*^*P* < 0.05, ^*∗∗*^*P* < 0.01, and ^*∗∗∗*^*P* < 0.001 (*n* = 8). Data were analyzed using SPSS software 26.0 (SPSS Inc., Chicago, USA); data represent the mean ± standard error of the mean. ^*∗*^*P* < 0.05, ^*∗∗*^*P* < 0.01, ^*∗∗∗*^*P* < 0.001, and ^*∗∗∗∗*^*P* < 0.0001.

After intervention, the left tibial plateau of each rat underwent HE and safranin-O staining, and the results are shown in Figures 1(a) and 1(b). Obviously, the cartilage surface of the ACLT group is damaged, with disorderly distributed and fewer chondrocytes, thinner cartilage layer, and incomplete tide line. However, the cartilage surface of the EA group was approximately intact, and the number of chondrocytes was generally consistent with that of the control group. The severity of OA was graded by the modified Mankin score (grades 0–14), as in Figure 1(c). Compared with the control group, the Mankin scores of the ACLT group and the EA group were significantly higher (*P* < 0.01), whereas the Mankin score of the EA group was significantly reduced (*P* < 0.01) in comparison to that of the ACLT group.

### 3.2. Effect of EA on the Morphology and Structure of Subchondral Bone in OA Rats

**Figure 2:**
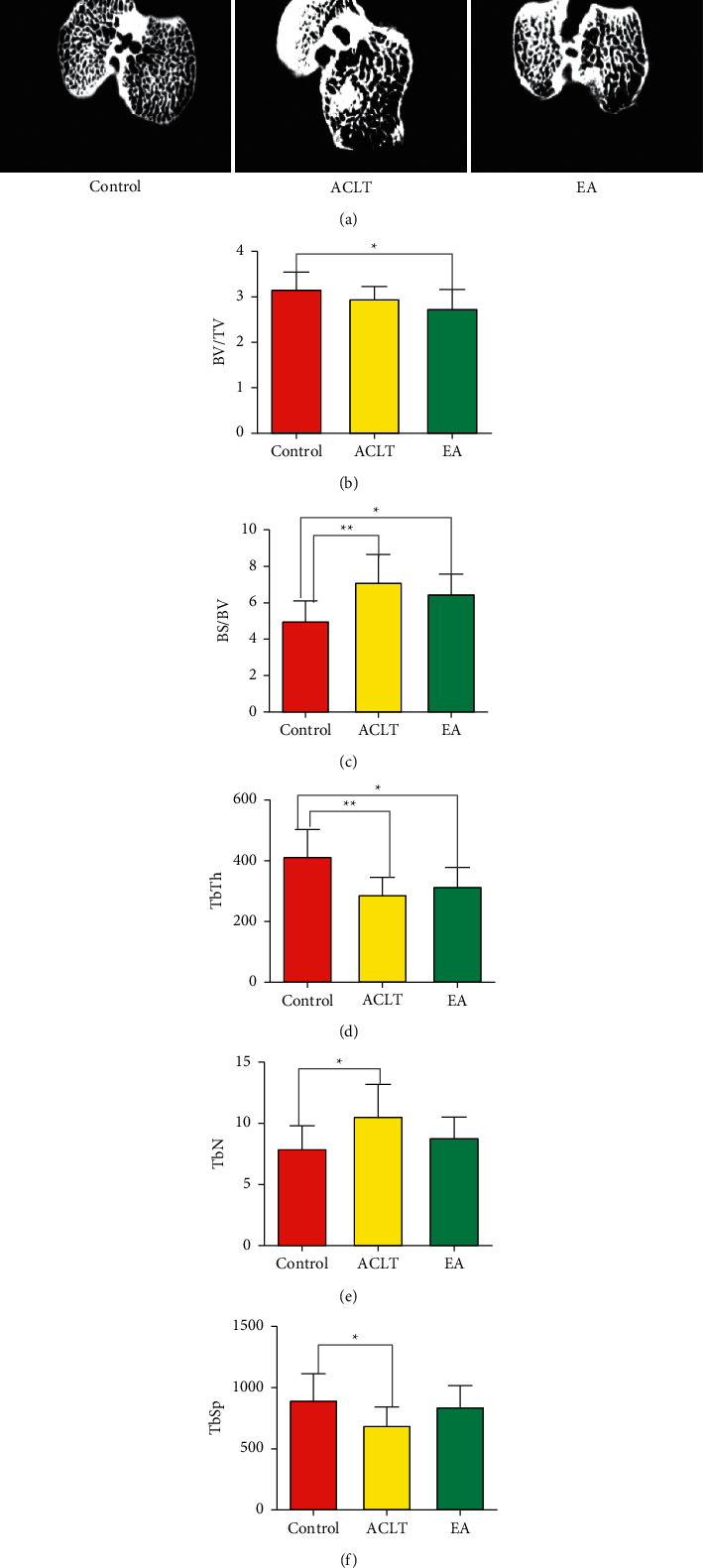
Representative micro-CT images of the right tibia. (a) Representative images. Bone parameters including (b) bone volume per tissue volume (BV/TV), (c) bone surface per bone volume (BS/BV), (d) trabecular bone thickness (Tb.Th), (e) trabecular number (Tb.N), and (f) trabecular separation (Tb.Sp) were assessed by quantitative micro-CT. Data were analyzed using SPSS software 26.0 (SPSS Inc., Chicago, USA); *n* = 8; data represent the mean ± standard error of the mean. ^*∗*^*P* < 0.05, *P* < 0.01, ^*∗∗∗*^*P* < 0.001, and ^*∗∗∗∗*^*P* < 0.0001.

**Figure 3:**
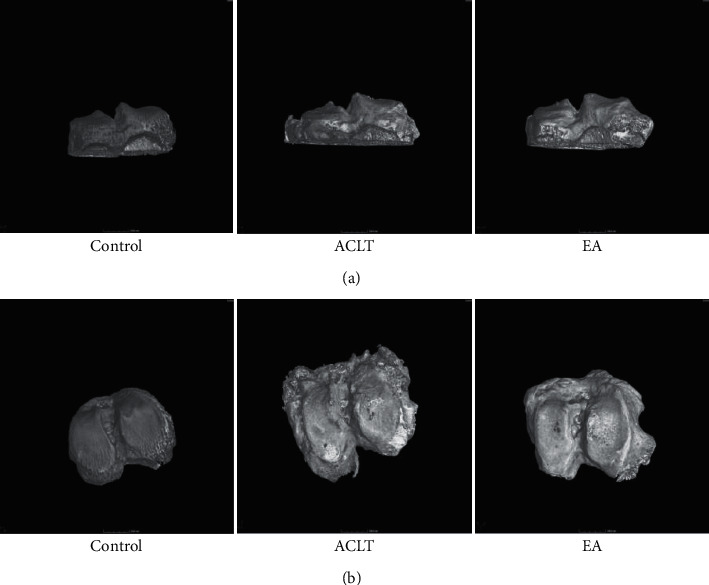
Representative 3D images of the right tibia. Control, ACLT, and EA, respectively, represent the control group, ACLT group, and EA group. (a), (b) The transversal section and coronal section.

Compared with the control group, local osteoporosis occurred in the ACLT group and the EA group in 3*μ*CT, but the overall bone gray value increased, as shown in Figure 2(a). Through quantitative analysis of the subchondral bone microstructure, compared with the control group, the BV/TV value of the ACLT group decreased, and the difference was not statistically significant (*P* > 0.05); consistently, the TbTh value of the ACLT group decreased compared to that of the control group, and the difference was statistically significant (*P* < 0.05); BS/BV, TbN, and TbSp are all increased in varying degrees, and the differences were statistically significant (*P* < 0.05), as shown in Figures 2(b)–2(f). Compared with the EA group, the BV/TV and BS/BV in the ACLT group increased at all levels, and the TbTh, TbN, and TbSp decreased to a variable extent, but the differences were not statistically significant (*P* > 0.05). Compared with the control group, the BV/TV and TbTh in the EA group were decreased, and the difference was statistically significant (*P* < 0.05). The TbSp was also decreased, but the difference was not statistically significant (*P* > 0.05). The BS/BV increased, and the difference was significant (*P* < 0.05). TbN increased, and the difference was not statistically significant (*P* > 0.05). The three-dimensional imaging in Figure 3 shows that compared with the control group, the cartilage surface defect and destruction occurred in the ACLT group and the EA group, with osteophyte formation. Compared with the ACLT group, the area of cartilage damage was smaller, and the number of osteophytes was smaller in the EA group. The results indicated that electroacupuncture may function as an effective treatment of osteoarthritis by regulating subchondral bone reconstruction.

### 3.3. EA Influences the Metabolism of Cartilage Matrix, SOX9, and HIF-A in OA Rats

**Figure 4:**
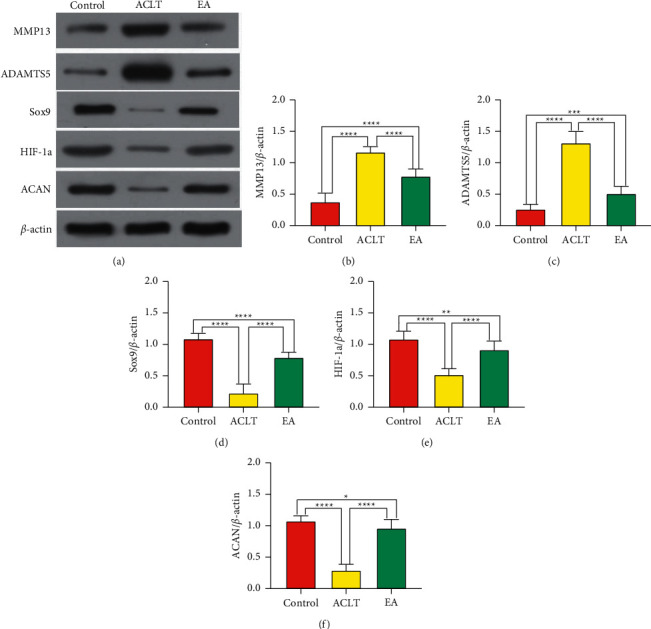
(a) Representative western blot analysis of p-P38, p-CREB, and MMP13 in cartilage from the three groups. The quantification analysis of (b) MMP13, (c) Adamts5, (d) Sox9, (e) HIF-1a, and (f) ACAN was conducted using SPSS software 26.0 (SPSS Inc., Chicago, USA) and normalized to *β*-actin. *n* = 8; data represent the mean ± standard error of the mean. ^*∗*^*P* < 0.05, ^*∗∗*^*P* < 0.01, ^*∗∗∗*^*P* < 0.001, and ^*∗∗∗∗*^*P* < 0.0001.

Lastly and most importantly, we want to know how electroacupuncture can delay cartilage degeneration. In Figure 4, western blotting shows that compared with the control group, the expression levels of HIF-1*α*, Sox9, and ACAN were significantly reduced, and the expression levels of MMP13 and ADAMTS5 were increased significantly in the ACLT group and the EA group (*P* < 0.05). Compared with ACLT, the expression levels of HIF-1*α*, Sox9, and ACAN increased significantly, while the expression levels of MMP13 and ADAMTS5 decreased in the EA group (*P* < 0.05). In conclusion, electroacupuncture for osteoarthritis is accomplished by upregulating the expression of HIF-1*α*, Sox9, and ACAN and downregulating the expression of MMP13 and ADAMTS5.

## 4. Discussion

OA, a chronic degenerative disorder, is prone to the elderly, which primarily presents with cartilage degeneration and subchondral bone defect [23]. There are multiple ways to induce OA, including surgical inductions such as ACLT, meniscal destabilization, chemical, and natural development. Respectively (monosodium iodoacetate), the MIA injection- and meniscal destabilization induced-OA model has been found to play the most critical role in evaluating knee pain [25]. Yet, natural development induced-OA is costly and time consuming, while ACLT is a standard OA induction procedure widely used in rats or rabbits to mimic posttraumatic OA in humans and leads to mild OA that limits to a partial cartilage degeneration [26]. In this study, we use safranin-O to value Mankin scores to verify successful establishment. In line with classical ACLT-induced posttraumatic OA, the rats in our study that experienced surgery showed typical posttraumatic features such as damaged cartilage surface, disorderly distribution, thinner cartilage layer, incomplete tide line, and fewer chondrocytes with higher Mankin scores than those of the control group, indicating that the cartilage was terribly damaged in rats [24].

Electroacupuncture (EA) is a nonpharmacological treatment basing on traditional acupuncture with electrical stimulation. A previous study demonstrated that EA has been proven effective in OA to relieve pain and eliminate inflammation with low adverse events and long-term efficiency [17]. Though there exist various therapies for OA, such as self-management, pharmacotherapy, repairment, and reconstruction, these therapies may accompany unavoidable side effects and relatively low effect [27]. Specifically, a prior study has showed that acupuncture could reach to the deeper cartilage to exert its function and may diminish inflammation and relieve pain in OA patients [28]. Additionally, our previous study has proved that EA can inhibit cartilage lesions in ACLT rats by downregulating mitogen-activated protein kinases (MAPKs) and MMP13 [29]. In this study, we found that EA decreased the Mankin scores in rats than ACLT group, which indicates that EA plays a critical role in cartilage protection and disease modification.

COL2a1 and ACAN, the major components of the cartilage extracellular matrix, act as a protective shield for cartilage. MMP13, a member of the metal matrix proteinases family, stimulates chondrocyte differentiation and induces chondrocyte hypertrophy, which promotes the progression of OA [30]. Additionally, the ADAMTS5 is a key enzyme leading to OA [31]. In our study, the expressions of ADAMTS5 and MMP13 in the ACLT group were higher than those in the control group, and ACAN decreased. COL2a1 and ACAN were increased in the EA group, accompanied by decreasing ADAMTS5 and MMP13 levels, suggesting that electroacupuncture can maintain the normal structure and function of cartilage by regulating matrix-degrading enzymes and extracellular matrix molecules of cartilage. In keeping with a prior study, the BS/BV, TbN, and TbSp in the ACLT group were increased, caused by uneven force after resection of the anterior cruciate ligament likely [23], while the electroacupuncture suppressed the certain changes in BS/BV, TbN, TbSp, and osteophyte, indicating that the electroacupuncture can also affect bone remolding effectively.

Interesting, the proteins such as SOX9 and HIF-1a were upregulated in rats after 12 weeks' EA treatment, which indicated that EA acts as a positive regulator for the two proteins. A prior study pointed out that SOX9 mediates adaptation to hypoxia in OA patients exhibiting aggressive cartilage disease [32]. Jordi mentioned that HIF-1a will be activated under a hypoxia environment [33]. Moreover, Chen noted that SOX9 and HIF-1a play an intimate role in cartilage via coimmunoprecipitation [34]. Thus, we pointed out that EA may alleviate OA by regulating SOX9 and HIF-1a in the hypoxia environment of cartilage. Sox9, closely related to bone diseases, is involved in a variety of stem cell differentiations and expressed in chondrocytes at different stages of mouse embryos [35]. Additionally, inflammatory factors inhibit Sox9 expression and promote cartilage degeneration, which indicated that enhancing Sox9 may promote cartilage formation and inhibit OA progression [34, 36]. Taking all results together, we suppose that EA could delay the cartilage degeneration and subchondral bone sclerosis to retard the development of OA, and this may relate to its upregulation of HIF-1a and SOX9. There are several limitations in this study. First, we did not use an inhibitor of HIF-1a or SOX9. Thus, it is not clear whether the two proteins act individually or in complexes. In addition, different intensities and pulses do not have the same effect, while we only set the fixed EA parameters such as intensity and pulse.

## 5. Conclusions

In this study, we point out that EA will delay the cartilage degeneration in the ACLT-induced OA models and increase the expression of proteins such as ACAN and COL2a1 which protect the cartilage. Also, we note that the upregulation of SOX9 and HIF-1a may act as the major mediation in the treatment. To some extent, this could provide evidence for EA clinical application. Regrettably, we still have a long way to find out the molecular mechanism and to test if there exists a direct correlation among the two proteins and electroacupuncture. In addition, the model of OA induced by ACLT stands only a type of OA, and it may be insufficient to apply to all kinds of OA. Thus, further investigations should be conducted to search.

## Data Availability

The research data used to support the findings of this study are included within the article.

## Abbreviations

OA: Osteoarthritis
KOA: Knee osteoarthritis
ACLT: Anterior cruciate ligament transection
HIF: Hypoxia-inducible factor
MMPs: Matrix metalloproteases
ADAMTS: Adisintegrin metalloproteinase with thrombospondin motifs
ACAN: Aggrecan
SOX: Sry-related HMG box
BV/TV: Bone volume fraction
Tb.Th: Trabecular thickness
Tb.N: Trabecular number
Tb.Sp: Trabecular separation
BS/BV: Bone surface/bone volume.
